# Innate sensing pathways: Defining new innate immune and inflammatory cell death pathways has shaped translational applications

**DOI:** 10.1371/journal.pbio.3002022

**Published:** 2023-02-10

**Authors:** Rebecca E. Tweedell, Sivakumar Prasanth Kumar, Thirumala-Devi Kanneganti

**Affiliations:** Department of Immunology, St. Jude Children’s Research Hospital, Memphis, Tennessee, United States of America

## Abstract

The past 20 years of research has revealed several new innate immune sensing and cell death pathways with disease relevance. This Perspective looks back over the impact these discoveries have had and forwards toward their future therapeutic potential.

## Teaser

The past 20 years of research has elucidated new innate immune sensing and cell death pathways with disease relevance. Future molecular characterization of these pathways and their crosstalk and functional redundancies will aid in development of therapeutic strategies.

This article is part of the *PLOS Biology* 20th Anniversary Collection.

**Fig 1:**
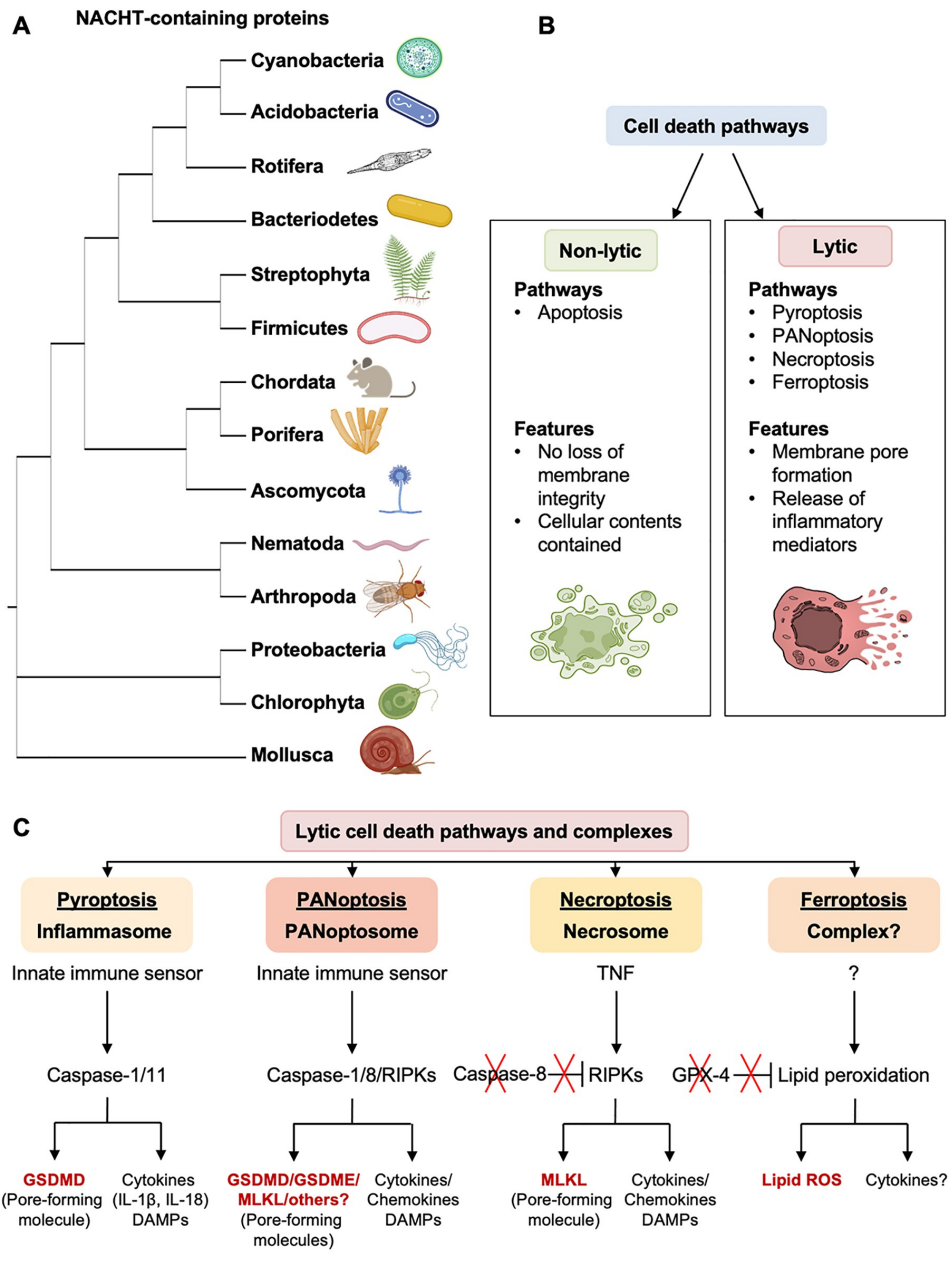
Evolutionary conservation of the innate immune pattern recognition receptor NLR family and the induction of III-RCD pathways. **(A)** Phylogenetic tree of NACHT domain-containing proteins, including the NLR family of pattern recognition receptors, from select bacterial and eukaryotic phyla. The tree was built using IQTREE2 phylogenetic software with protein sequences from Mollusca (*Candidula unifasciata*), Proteobacteria (*Pseudomonas fluorescens*), Nematoda (*Caenorhabditis elegans*), Arthropoda (*Drosophila melanogaster*), Chlorophyta (*Chlamydomonas reinhardtii*), Ascomycota (*Aspergillus niger*), Streptophyta (*Selaginella moellendorffii*), Chordata (*Mus musculus*), Porifera (*Amphimedon queenslandica*), Firmicutes (*Roseburia inulinoivorans*), Rotifera (*Adineta ricciae*), Bacteriodetes (*Chryseobacterium oleae*), Cyanobacteria (*Microcystis aeruginosa*), and Acidobacteria (*Luteitalea pratensis*). **(B)** Cell death pathways can be divided into lytic and nonlytic categories, with distinct features and pathways in each. **(C)** A simplified schematic representation of lytic cell death pathway mechanisms. Inflammasomes (pyroptosis), PANoptosomes (PANoptosis), and necrosomes (necroptosis) induce lytic regulated cell death pathways. Another recently described form of lytic cell death, ferroptosis, does not have a specific cell death-inducing complex identified to date. In PANoptosomes, crosstalk and redundancies between caspase-1, caspase-8, and/or RIPK molecules can occur to drive cell death. Pyroptosis and PANoptosis depend on the activation of caspases, while necroptosis depends on the inhibition of caspase-8. In contrast, ferroptosis occurs in response to GPX-4 inhibition. DAMPs, damage-associated molecular patterns; GPX-4, glutathione peroxidase 4; GSDMD, gasdermin D; GSDME, gasdermin E; III-RCD, innate immune inflammatory regulated cell death; IL, interleukin; MLKL, mixed lineage kinase domain-like; NLR, NOD-like receptor; RIPK, receptor interacting protein kinase; ROS, reactive oxygen species; TNF, tumor necrosis factor. Figure created with components from BioRender.

Innate immunity provides the first line of defense against disease. To carry out this critical function, the innate immune system is made up of sensor molecules called pattern recognition receptors (PRRs) that detect pathogen-associated and damage-associated molecular patterns (PAMPs and DAMPs) to initiate signaling pathways that activate a broader immune response and regulated cell death (RCD). There are several families of PRRs, including Toll-like receptors (TLRs), NOD-like receptors (NLRs), and RIG-I-like receptors (RLRs), and they are highly evolutionarily conserved (Fig 1A). For instance, mammalian NLRs were discovered to have structural similarity to plant NLRs [1], and the origins of mammalian cGAS–STING machinery were recently traced back to bacteria [2]. Similarly, many sensing and signaling strategies are conserved across genera, such as the ability of NLRs to act as both direct sensors and helper molecules in plants, which is beginning to be appreciated in mammals as well [3]. This evolutionary conservation highlights the critical functions of these molecules for organismal survival.

Because of their essential and multifaceted functions in health and disease, innate immune sensors and their downstream pathways have been a major focus of recent research. The advent of new technologies and genetic tools to study the immune system has greatly advanced this field and enabled cutting-edge breakthroughs in our understanding of innate immune-mediated cell death. Until approximately 20 years ago, apoptosis was the only widely accepted form of RCD, and it was thought to be a developmental process that allowed nonlytic, immunologically silent clearance of cells that were no longer needed. There was a gradual shift in this understanding as studies began to show that infections could also cause RCD [4]. But this death was not silent; instead, it was lytic and inflammatory, resulting in the release of proinflammatory cytokines and signaling molecules (Fig 1B). As the momentum in the innate immunity and cell death fields began to bring these previously divided sectors of research together, it became clear that apoptosis was not the only RCD pathway. This led to descriptions of many new cell death pathways and their molecular mechanisms. In 2001, the innate immune inflammatory RCD (III-RCD) pathway “pyroptosis” was named, followed in 2002 by the description of its multiprotein cell death complex known as the “inflammasome” [5]; this was shortly followed by the identification of other lytic RCD pathways, including “necroptosis” in 2005 [6], “ferroptosis” in 2012 [7] (Fig 1C), and several others [8].

Building upon these findings, the innate immune sensing and cell death fields have grown exponentially. As a prime example of this growth, there were only 92 publications in PubMed on pyroptosis in its first 10 years of study (2001 to 2011), but there were 2,204 just last year (2022). As a result of the increased mechanistic understanding of RCD, cell death molecules are now widely implicated across the disease spectrum, from infections and autoinflammatory diseases to cancers, and these molecules are prime targets for drug development [9]. However, few clinical trials using therapeutics targeting cell death machinery have been successful to date, and much work remains to optimize treatment strategies.

Clinical translation continues to be a top priority in innate immunity and cell death fields, leading investigators to ask fundamental questions about RCD pathways to improve therapeutic strategies. As research in these areas has grown, so too has the understanding that there are mechanistic connections and functional redundancies among multiple forms of RCD. This may be one of the key factors influencing the lack of clinical effectiveness for therapeutics targeting cell death machinery, such as caspases and inflammasome components [9]. Studies of this crosstalk have suggested a paradigm shift in the field and provided multiple lines of evidence connecting RCD pathways that were historically viewed as independent. These findings have highlighted an important gap in our mechanistic understanding of RCD and innate immune pathway components.

Recent progress to fill this gap has identified regulatory mechanisms controlling crosstalk between RCD pathways and the central roles of caspases in these connections. Caspase-1 was characterized as a component of the inflammasome and pyroptosis, but it can also cleave apoptotic substrates, including caspase-7 and PARP [10]. Additionally, functional redundancies have been found between caspase-1 and caspase-8 in disease mechanisms [11]. Caspase-8 has long been known to be an initiator of apoptosis and an inhibitor of necroptosis, but work in the past decade found that caspase-8 also regulates the NLRP3 inflammasome and pyroptosis [12]. These findings connecting caspase-8, caspase-1, and multiple RCD pathways led to the identification of new multiprotein cell death complexes, called “PANoptosomes”, which regulate the unique III-RCD pathway known as “PANoptosis” (Fig 1C) [10].

PANoptosomes form when innate immune sensors detect pathogens, PAMPs, DAMPs, homeostatic perturbations, or the cytokines produced downstream. The first PANoptosome identified is formed by Z-DNA binding protein 1 (ZBP1), an innate immune sensor that detects influenza A virus to activate the NLRP3 inflammasome and PANoptosis [3]. To date, three distinct PANoptosome complexes have been identified, with key conserved molecules, such as caspase-8, found in each [10]. These PANoptosomes use different sensors to respond to specific perturbations, which is similar to how inflammasomes form. Additionally, PANoptosis has been observed in many diseases, including infections, inflammatory diseases, and cancers, where it can have detrimental or beneficial effects depending on the specific disease context [10]. For example, in cytokine storm-related clinical pathology in COVID-19 and other diseases, PANoptosis has a disease-exacerbating effect [13]. By contrast, PANoptosis can be induced through the same mechanism to inhibit tumor growth in murine xenograft tumor models [10]. Therefore, it will be critical to mechanistically characterize this III-RCD pathway in disease to optimize therapeutic strategies.

Overall, the fields of innate immune sensing and cell death have progressed extensively in the past 20 years, highlighting key molecular similarities and differences between RCD pathways. Looking to the future, work to improve the translational application of these discoveries is critically important. Current molecular evidence suggests that many disease phenotypes cannot be explained by previously identified RCD pathways individually [10]. Future studies will need to view RCD through the lens of multiple interconnected pathways with functional redundancies and crosstalk (as PANoptosis does) to enable researchers to consider the full suite of molecular components involved and identify unique therapeutic targets. Such an approach would also help to address why inhibiting a single molecule may not be sufficient to prevent RCD and inflammation.

Future work to identify new therapeutic targets in innate immunity and cell death complexes and to translate these findings to the clinic should leverage the power of new technological advancements. The advent of CRISPR–Cas9, single-cell analysis technologies, advanced imaging capabilities, and cryo-EM have already rapidly accelerated progress in the field and paved the way for continued discovery. It has never been a better time to investigate both the evolutionarily conserved and distinct aspects of innate immune signaling and cell death pathways in health and disease to translate these mechanistic findings into therapeutic strategies.

## Abbreviations

DAMP: damage-associated molecular pattern
III-RCD: innate immune inflammatory RCD
NLR: NOD-like receptor
PAMP: pathogen-associated molecular pattern
PRR: pattern recognition receptor
RCD: regulated cell death
RLR: RIG-I-like receptor
TLR: Toll-like receptor
ZBP1: Z-DNA binding protein 1
